# Chemotherapy backbone and outcomes of immune checkpoint inhibitor combinations across solid tumors: a pan-tumor network meta-analysis

**DOI:** 10.1093/oncolo/oyag365

**Published:** 2026-09-17

**Authors:** Luisana Sisca, Mariam Grazia Polito, Alessio Cortellini, Marco Russano, Marianna Silletta, Roberta Scafetta, Silvia Calagna, Michele Iuliani, Silvia Cavaliere, Sonia Simonetti, Bruno Vincenzi, Giuseppe Tonini, Francesco Pantano

**Affiliations:** Department of Medical Oncology, Fondazione Policlinico Universitario Campus Bio-Medico, Rome, 00128, Italy; Department of Translational Research, Institut Curie, Paris, 75005, France; Department of Medical Oncology, Fondazione Policlinico Universitario Campus Bio-Medico, Rome, 00128, Italy; Territorial Oncology Unit, Sapienza University of Rome, Aprilia, 04011, Italy; Department of Medical Oncology, Fondazione Policlinico Universitario Campus Bio-Medico, Rome, 00128, Italy; Department of Surgery and Cancer, Imperial College London, London, W12 0NN, United Kingdom; Department of Medical Oncology, Fondazione Policlinico Universitario Campus Bio-Medico, Rome, 00128, Italy; Department of Medical Oncology, Fondazione Policlinico Universitario Campus Bio-Medico, Rome, 00128, Italy; Department of Medical Oncology, Fondazione Policlinico Universitario Campus Bio-Medico, Rome, 00128, Italy; Department of Medical Oncology, Fondazione Policlinico Universitario Campus Bio-Medico, Rome, 00128, Italy; Department of Medical Oncology, Fondazione Policlinico Universitario Campus Bio-Medico, Rome, 00128, Italy; Department of Medical Oncology, Fondazione Policlinico Universitario Campus Bio-Medico, Rome, 00128, Italy; Department of Medical Oncology, Fondazione Policlinico Universitario Campus Bio-Medico, Rome, 00128, Italy; Department of Medical Oncology, Fondazione Policlinico Universitario Campus Bio-Medico, Rome, 00128, Italy; Department of Medical Oncology, Fondazione Policlinico Universitario Campus Bio-Medico, Rome, 00128, Italy; Department of Medical Oncology, Fondazione Policlinico Universitario Campus Bio-Medico, Rome, 00128, Italy

**Keywords:** immune checkpoint inhibitors, chemotherapy backbone, network meta-analysis, chemo-immunotherapy, solid tumors, immunotherapy

## Abstract

**Background:**

Immune checkpoint inhibitors (ICIs) combined with chemotherapy represent a first-line standard across multiple solid tumors; however, the contribution of chemotherapy backbone to the efficacy of chemo-immunotherapy remains uncertain.

**Methods:**

We conducted a systematic review and network meta-analysis of phase II-III randomized trials comparing ICI–chemotherapy vs chemotherapy alone, with the aim of evaluating whether chemotherapy backbone selection was associated with differences in clinical outcomes across tumor types.

**Results:**

Thirty-nine trials including over 30 000 patients were analyzed. Detailed characteristics of included trials by tumor type are reported in Tables S1-S6. Chemo–immunotherapy significantly improved progression-free survival (hazard ratio [HR] 0.69, 95% CI 0.66-0.72) and overall survival (HR 0.78, 95% CI 0.76-0.81). Among the evaluated chemotherapy backbones, platinum-pemetrexed was associated with the greatest relative survival benefit (HR 0.66), followed by platinum–taxane and fluoropyrimidine–platinum regimens (HR 0.78), whereas platinum–gemcitabine showed comparatively lower benefit (HR 0.83). Anti–PD-1 regimens demonstrated numerically greater benefit than anti–PD-L1 therapies, although these findings should be interpreted in light of the non-random distribution of treatment regimens across tumor types. Because chemotherapy backbones were closely linked to tumor-specific standards of care, these differences cannot be interpreted as evidence of superiority across tumor types. All combinations increased grade ≥3 adverse events.

**Conclusion:**

Differences in outcomes were observed across chemotherapy backbones; however, these findings likely reflect the combined influence of tumor-specific treatment paradigms, underlying disease biology, and potentially the chemotherapy backbone itself. Therefore, the results should be considered hypothesis-generating and warrant prospective validation within individual tumor types.

Implications for practice This pan-tumor network meta-analysis suggests that chemotherapy backbone selection may contribute to differences in efficacy of immune checkpoint inhibitor–chemotherapy combinations across solid tumors. Differences in relative treatment benefit were observed across chemotherapy backbones; however, these findings should be interpreted cautiously because chemotherapy regimens are closely linked to tumor-specific standards of care and underlying disease biology. Rather than supporting the superiority of one chemotherapy backbone over another across cancers, these findings provide a rationale for prospective evaluation of chemotherapy backbone selection within individual tumor types.

## Introduction

The introduction of immune checkpoint inhibitors has profoundly changed the therapeutic landscape of solid tumors, establishing chemo–immunotherapy as a standard first-line option in several malignancies. Despite this success, the magnitude of benefit varies widely among tumor types, and the reasons underlying this variability remain only partially understood.^1^ Much of the difference has traditionally been attributed to intrinsic biological characteristics of individual cancers, including PD-L1 expression. Yet mounting evidence suggests that chemotherapy itself may contribute more substantially to shaping the efficacy of immunotherapy than previously appreciated.^2^

Chemotherapeutic agents differ markedly in their capacity to induce immunogenic cell death, enhance antigen presentation, modulate dendritic cell function, deplete suppressive myeloid populations, or favor T-cell activation.^3^ Taxanes, platinum compounds, antimetabolites, and other cytotoxic agents possess distinct immunomodulatory properties, raising the possibility that the chemotherapy backbone used alongside immunotherapy may contribute to differences in treatment outcomes.^4^ For decades, the selection of chemotherapy in clinical trials has generally followed historical, disease-specific standards rather than considerations related to immunologic synergy. As a consequence, the relative contribution of specific backbones to the success of chemo-immunotherapy combinations remains insufficiently characterized.^5^

The availability of numerous phase II–III randomized trials across multiple tumor types provides an opportunity to explore whether consistent patterns of association exist between chemotherapy backbone and the efficacy of chemo-immunotherapy. Although cross-tumor comparisons cannot disentangle the contribution of chemotherapy backbone from the underlying biology of the diseases in which these regimens are used, they may generate hypotheses regarding treatment strategies that warrant further evaluation within individual tumor types.^6^

To address this, we conducted the first comprehensive pan-tumor network meta-analysis of phase II-III randomized trials evaluating chemo–immunotherapy vs chemotherapy alone. By integrating 39 randomized trials including more than 30 000 patients, this study aimed to investigate whether different chemotherapy backbones are associated with differential clinical outcomes when combined with immune checkpoint inhibitors across solid tumors. In doing so, we sought to evaluate whether chemotherapy backbone selection is associated with differential outcomes when combined with immune checkpoint inhibitors and whether these patterns are consistent across different tumor types. Given the close relationship between chemotherapy backbone and tumor-specific standards of care, our objective was not to establish the superiority of individual regimens across malignancies, but rather to explore whether consistent patterns of association could be identified across the available randomized evidence while acknowledging that such associations cannot establish causality or support cross-tumor interchangeability of chemotherapy regimens.^7^ Accordingly, this study should be viewed as an exploratory, hypothesis-generating analysis intended to inform future disease-specific investigations rather than to redefine existing standards of care. Importantly, because chemotherapy backbones are intrinsically linked to tumor-specific treatment paradigms, the present analysis was not designed to disentangle the independent contribution of chemotherapy from the underlying biology of individual malignancies. Rather, it was intended to identify reproducible patterns of association that may help generate hypotheses for future within-disease comparative studies.

## Methods

This systematic review and network meta-analysis was performed according to PRISMA-NMA guidelines (Figure 1) and was prospectively registered in PROSPERO (registration ID CRD420251167238). A comprehensive search of PubMed, Embase, the Cochrane Library, major oncology conference proceedings, and regulatory submissions was conducted through June 2025 to identify phase II-III randomized controlled trials evaluating immune checkpoint inhibitors combined with chemotherapy as first-line treatment for advanced or metastatic solid tumors.

**Figure 1.**
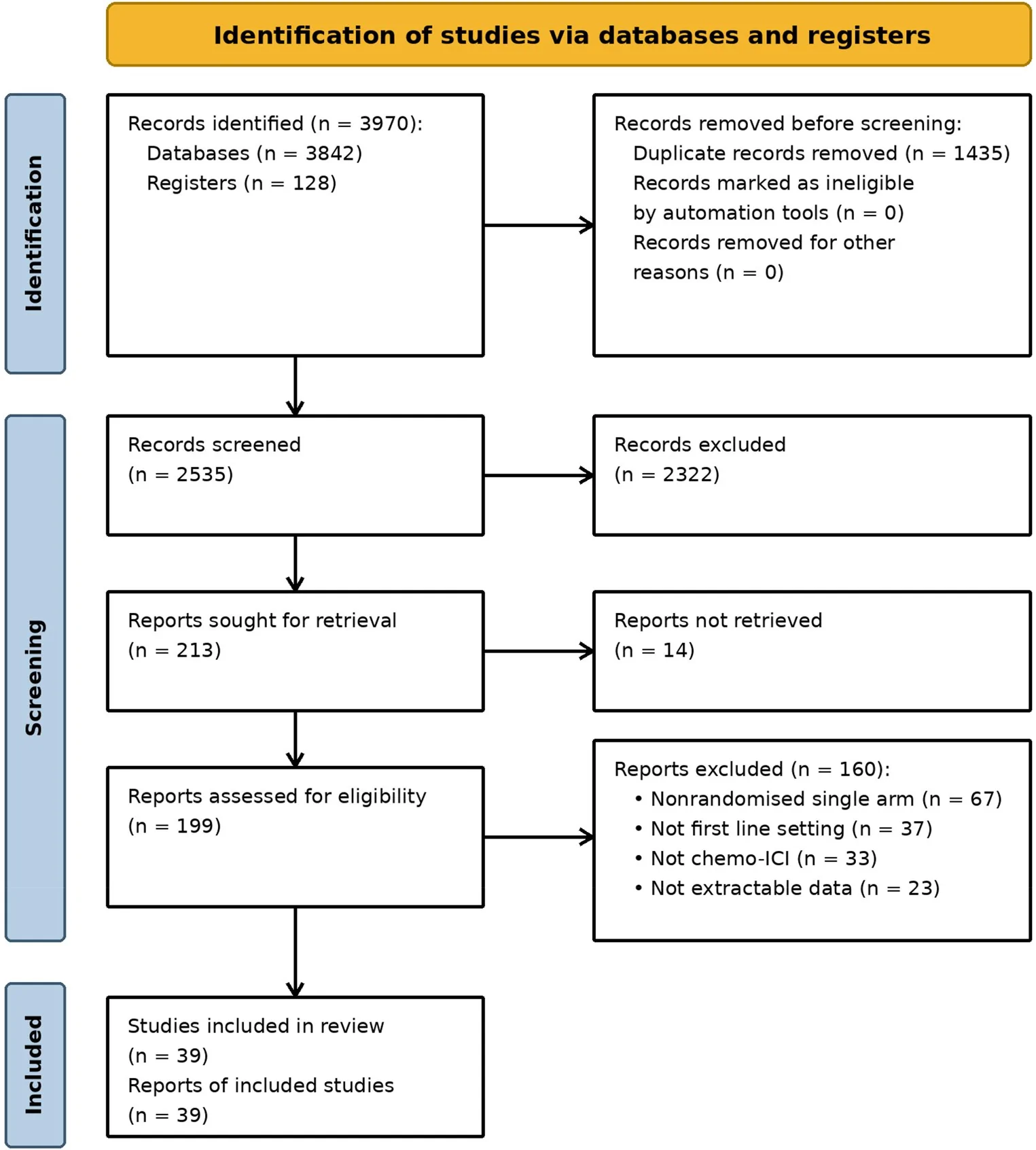
PRISMA 2020 flow diagram. Summary of the study selection process leading to the inclusion of 39 randomized controlled trials.

The core PubMed search strategy was:

(“pembrolizumab” OR “nivolumab” OR “cemiplimab” OR “atezolizumab” OR “durvalumab” OR “avelumab” OR “anti–PD-1” OR “anti–PD-L1” OR “immune checkpoint inhibitor*”)

AND

(“chemotherapy” OR “taxane*” OR “paclitaxel” OR “docetaxel” OR “platinum” OR “carboplatin” OR “cisplatin” OR “gemcitabine” OR “pemetrexed” OR “fluorouracil”)

AND

(“first-line” OR “front-line” OR “treatment-naïve” OR “advanced” OR “metastatic”)

AND

(“phase III” OR “phase II” OR “randomized controlled trial” OR “randomised controlled trial” OR “RCT”).

Equivalent strings were adapted for Embase and Cochrane using Emtree and CENTRAL syntax.

Two reviewers (L.S. and M.G.P.) independently performed the study selection process. Titles, abstracts, and full-texts were screened in duplicate, with disagreements resolved through discussion and consensus. Trials were included if they compared an anti–PD-1 or anti–PD-L1 agent plus chemotherapy with a chemotherapy-alone control arm. Studies conducted exclusively in the adjuvant or neoadjuvant setting were excluded.

Data extraction encompassed tumor type, immunotherapy agent and class, chemotherapy regimen, sample size, and published effect estimates for overall survival, progression-free survival, and grade ≥3 adverse events. Chemotherapy backbones were classified into predefined groups based on pharmacologic and clinical relevance: platinum–pemetrexed, platinum–taxane, taxane monotherapy, fluoropyrimidine–platinum, and platinum–gemcitabine. Additional backbones were analyzed when supported by sufficient data. When tumor-specific datasets were available, they were analyzed separately to obtain disease-level estimates.

All statistical analyses were conducted in R, following the pipeline implemented in the original analysis script. The odds ratio (OR) for any grade ≥3 adverse events was pooled for meta-analysis using random or fixed effects models based on detected heterogeneity. A meta-analysis of efficacy data was also performed, and hazard ratios (HRs) were pooled for progression-free survival (PFS) and overall survival (OS).

Heterogeneity among studies was assessed using the *I*² statistic. Funnel plots were used to assess publication bias and Egger’s test was performed when applicable, depending on the number of included studies.

In addition, a network meta-analysis was conducted, grouping studies according to the chemotherapy backbone, the mechanism of action of ICIs, and tumor primitivity to enable comparative evaluation across these predefined subgroups. The network meta-analysis was conducted within a frequentist framework using a random-effects consistency model implemented in the netmeta package in R. Treatment effects were expressed as log HRs and pooled using inverse-variance weighting. Between-study heterogeneity was quantified using τ². Network consistency was evaluated using the design-by-treatment interaction model. Treatment ranking was estimated using P-scores. The assumption of transitivity underlying the network meta-analysis was considered clinically plausible, as all included trials evaluated first-line chemo–immunotherapy vs chemotherapy alone in advanced solid tumors, using comparable endpoints (OS and PFS), similar effect measures (HRs), and broadly analogous patient populations in terms of treatment-naïve advanced disease setting. Nevertheless, given the nonrandom distribution of chemotherapy backbones across tumor types, this assumption should be interpreted with appropriate caution when comparing treatments across different malignancies.

Risk of bias for each trial was assessed independently by two reviewers using the Cochrane RoB-2 tool, evaluating randomization, deviations from intended interventions, missing outcome data, measurement of outcomes, and selective reporting.

All data extracted for this meta-analysis are derived from published trials and are available in the Supplementary Material or upon reasonable request.

## Results

Thirty-nine phase II–III randomized controlled trials^8–46^ enrolling more than 30 000 patients were included in the network meta-analysis. One multi-cohort study was analyzed as separate backbone-specific comparisons. The studies spanned breast, gastrointestinal (including esophageal), genitourinary, lung, and gynecologic cancers. Urogenital studies evaluated anti–PD-1/PD-L1 agents (pembrolizumab, nivolumab, atezolizumab) in first-line urothelial carcinoma, consistently combined with gemcitabine–platinum chemotherapy. In lung cancer, anti–PD-1/PD-L1 monotherapy or nivolumab–ipilimumab was paired with platinum doublets, using pemetrexed for nonsquamous histology and gemcitabine or taxane-based regimens for squamous tumors. Gynecologic trials mainly investigated anti–PD-1/PD-L1 agents (avelumab, atezolizumab, dostarlimab, and pembrolizumab) in combination with carboplatin–paclitaxel. Gastrointestinal studies used anti–PD-1/PD-L1 inhibitors (nivolumab, pembrolizumab, tislelizumab, sintilimab, and sugemalimab) with fluoropyrimidine–platinum, or cisplatin–gemcitabine, while some trials incorporated dual checkpoint blockade with an anti-CTLA-4 agent. Breast cancer trials assessed anti–PD-1/PD-L1 regimens, mainly atezolizumab or pembrolizumab, combined with taxane-based chemotherapy (paclitaxel, nab-paclitaxel) or gemcitabine–carboplatin.

Risk-of-bias assessment across trials is shown in Figure S1 (see online supplementary material for a color version of this figure).

Median OS and PFS gains were consistent with the HR-based findings. Across the included trials, chemo–immunotherapy increased median PFS and OS compared with chemotherapy alone, with absolute improvements generally ranging from modest to clinically meaningful depending on tumor type and chemotherapy backbone. Regimens such as platinum–pemetrexed or platinum–taxane showed some of the largest absolute gains, while platinum–gemcitabine and cyclophosphamide-based combinations demonstrated smaller differences.

Across all eligible trials, chemo–immunotherapy significantly improved progression-free survival (HR 0.69; 95% CI 0.66–0.72; I² = 54%; Figure S2) and overall survival (HR 0.78; 95% CI 0.76–0.81; Figure 2). However, the magnitude of benefit varied substantially across chemotherapy backbones, tumor types, and immunotherapy classes.

**Figure 2.**
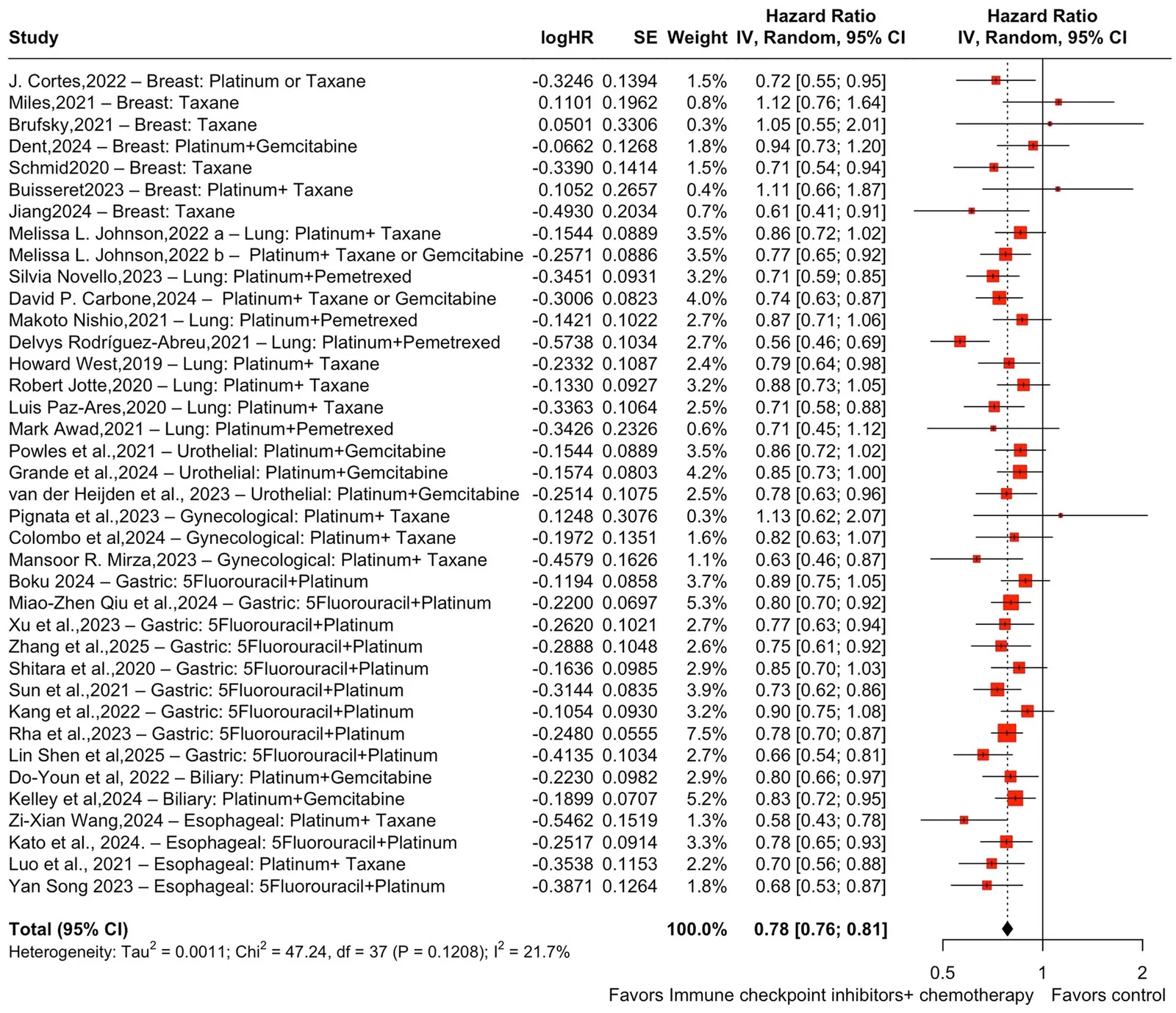
Overall survival with first-line chemo-immunotherapy across all included trials. Forest plot displaying individual and pooled hazard ratios comparing chemo-immunotherapy vs chemotherapy alone. Overall pooled effect: *P* < .0001. Abbreviations: ICIs = immune checkpoint inhibitors; HR = hazard ratio.

At the backbone level, platinum–pemetrexed was associated with the largest relative overall survival benefit (HR 0.66; 95% CI 0.58-0.75), followed by fluoropyrimidine–platinum and platinum–taxane regimens (HR 0.78 each), taxane monotherapy (HR 0.80), and platinum–gemcitabine (HR 0.83), although these associations should not be interpreted as comparative evidence of backbone superiority because chemotherapy regimens were closely linked to tumor type across the included trials (Figure 3). Cyclophosphamide-based combinations did not demonstrate a statistically significant survival advantage. Although numerical differences across chemotherapy backbones were observed, formal indirect comparisons between regimens should be interpreted cautiously given overlapping confidence intervals and the potential confounding effect of tumor-type distribution across studies. Progression-free survival analyses demonstrated a consistent gradient across chemotherapy backbones (Figure S3, see online supplementary material for a color version of this figure). Platinum–pemetrexed showed the lowest HR (HR 0.44; 95% CI 0.39-0.51), followed by taxane monotherapy (HR 0.51; 0.42-0.62) and platinum–taxane combinations (HR 0.55; 0.49-0.60). Fluoropyrimidine–platinum regimens were associated with an HR of 0.58 (0.53-0.63), whereas platinum–gemcitabine displayed a comparatively attenuated benefit (HR 0.67; 0.59-0.75). Cyclophosphamide-based regimens showed limited precision due to wide confidence intervals.

**Figure 3.**
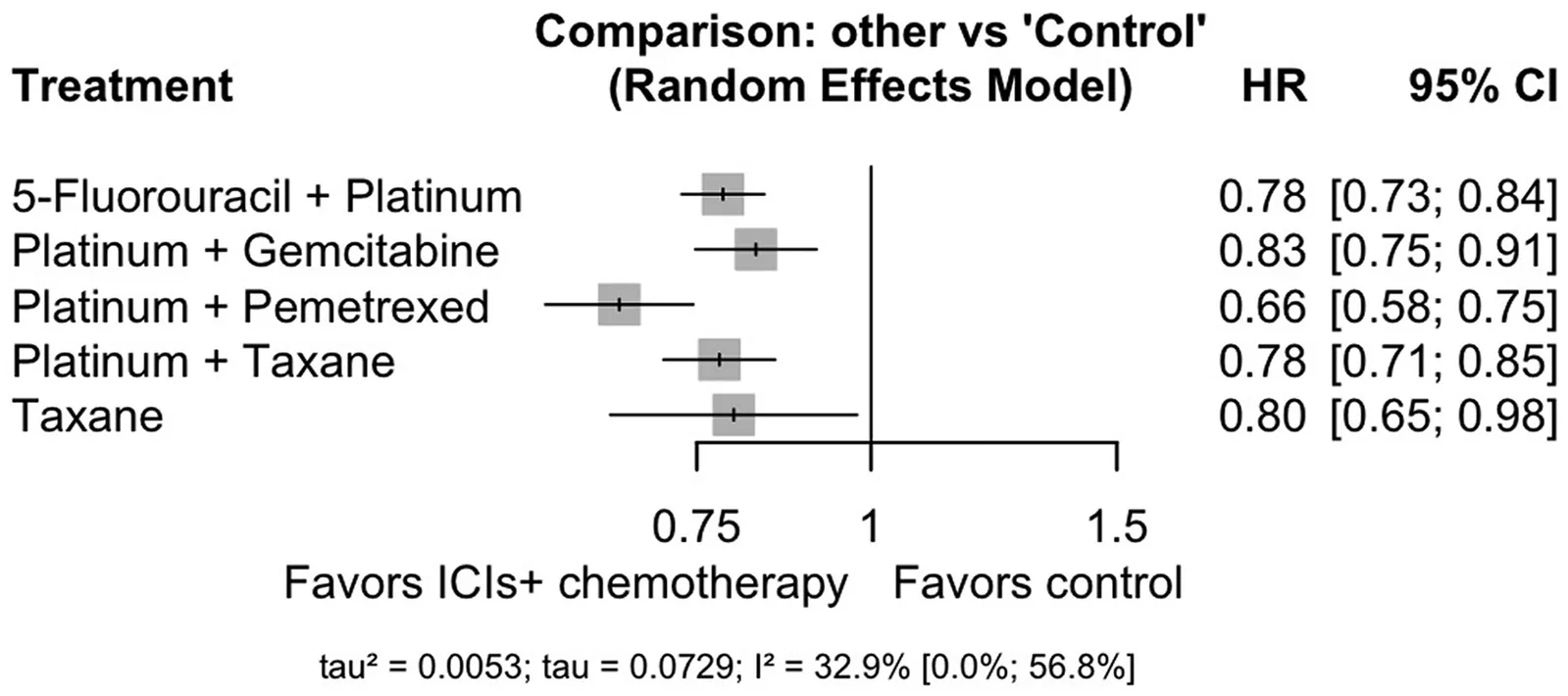
Forest plot of overall survival stratified by chemotherapy backbone. Forest plot comparing pooled OS hazard ratios across predefined chemotherapy backbones combined with immune checkpoint inhibitors. *I*² = 32.9%, indicating moderate between-study heterogeneity. Platinum-pemetrexed (*k* = 4), platinum-taxane (*k* = 10), fluoropyrimidine-platinum (*k* = 11), platinum-gemcitabine (*k* = 5), and taxane-only regimens (*k* = 4) were included in the analysis. Abbreviations: ICIs = immune checkpoint inhibitors; HR = hazard ratio.

Stratified analyses of individual chemo-immunotherapy combinations confirmed consistent benefit across regimens (Figure S4 [see online supplementary material for a color version of this figure] for PFS; Figure S5 [see online supplementary material for a color version of this figure] for OS).

Disease-specific analyses reproduced these trends across tumor types (Figure S6 [see online supplementary material for a color version of this figure] for PFS; Figure S7 [see online supplementary material for a color version of this figure] for OS). In lung cancer, platinum–pemetrexed and platinum–taxane demonstrated HRs of 0.45 (0.38-0.52) and 0.56 (0.48-0.65), respectively. Gastrointestinal malignancies showed robust improvements with fluoropyrimidine–platinum (0.57; 0.51-0.63), while esophageal cancers particularly benefited from platinum–taxane (0.50; 0.39-0.64), followed by fluoropyrimidine–platinum (0.60; 0.47-0.75). Breast cancer trials demonstrated benefit with taxane monotherapy (0.51; 0.41-0.62), comparable to platinum–taxane combinations.

The role of immune checkpoint inhibitors in treatment benefit was also investigated, and this class of agents showed a relevant influence on outcomes across analyses. Anti–PD-1 agents showed numerically greater pooled survival benefit compared with anti–PDL-1 agents (Figure S8 [see online supplementary material for a color version of this figure] for PFS; Figure S9 [see online supplementary material for a color version of this figure] for OS).; however, meta-regression analysis did not demonstrate a statistically significant class-level effect, and therefore these differences should be interpreted as exploratory. Given the nonrandom distribution of anti–PD-1 and anti–PDL-1 agents across tumor types and treatment settings, these subgroup analyses remain potentially influenced by underlying disease-specific characteristics. When stratified by chemotherapy backbone, anti–PD-1 regimens showed a consistent numerical trend toward greater benefit across most combinations, with the most pronounced differences in PFS observed for taxane-based therapies, where anti–PD-1 combinations achieved an HR of 0.50 (0.44-0.57) vs 0.57 (0.50-0.65) for anti–PDL-1. Similar trends were observed for OS.

Cross-class and cross-backbone analyses further confirmed these trends. Under anti–PD-1 treatment, the best-performing regimens were platinum–pemetrexed (0.65; 0.57-0.74) and platinum–taxane (0.72; 0.63-0.81), closely followed by fluoropyrimidine–platinum (0.80; 0.74-0.87) and platinum–gemcitabine (0.82; 0.73-0.92). Anti–PDL-1 regimens demonstrated weaker but still meaningful activity, with HRs ranging from 0.68 (0.56-0.84) for fluoropyrimidine–platinum to 0.81 (0.71-0.93) for platinum–taxane and 0.85 (0.68-1.06) for platinum–gemcitabine. Taxane monotherapy showed comparable efficacy between anti–PD-1 and anti–PDL-1 agents in OS (HR 0.51 vs 0.50), while modest differences were observed in PFS. Taken together, these results suggest a numerical pattern of relative treatment benefit across chemotherapy backbones. However, because chemotherapy regimens were not randomly distributed across tumor types, these observations cannot distinguish backbone-specific effects from underlying disease biology and should therefore be considered exploratory. These patterns appeared directionally consistent across tumor types, although formal meta-regression did not identify statistically significant moderators. Across all eligible trials, chemo–immunotherapy was associated with a modest increase in grade ≥3 adverse events compared with chemotherapy alone. As shown in Figure S10 (see online supplementary material for a color version of this figure), the magnitude of toxicity varied across tumor types, ranging from OR 1.18 in gynecologic cancers to OR 1.43 in esophageal cancer. Breast and lung cancers showed increased toxicity (OR 1.37 and 1.46, respectively), while urothelial and biliary cancers demonstrated more moderate excess toxicity.

Class-level comparisons (Figure S11, see online supplementary material for a color version of this figure) revealed slightly higher toxicity with anti–PDL-1 regimens compared with anti–PD-1 agents, although both classes displayed consistent safety profiles aligned with previous randomized trials.

When stratified by chemotherapy backbone (Figure 4), the highest odds ratios were observed with taxane monotherapy (OR 1.47) and platinum–pemetrexed combinations (OR 1.45), followed by platinum–taxane (OR 1.31) and fluoropyrimidine–platinum regimens (OR 1.38). Platinum–gemcitabine showed a comparatively lower toxicity increase (OR 1.23), while cyclophosphamide-based regimens did not demonstrate a significant excess risk. No new or unexpected safety signals emerged from the pooled analysis. We additionally conducted a meta-regression analysis to explore whether study-level variables acted as effect modifiers. The inclusion of tumor type and immunotherapy class as covariates did not identify any statistically significant associations with the outcome of interest (Figures S12 and S13, see online supplementary material for a color version of these figures).

**Figure 4.**
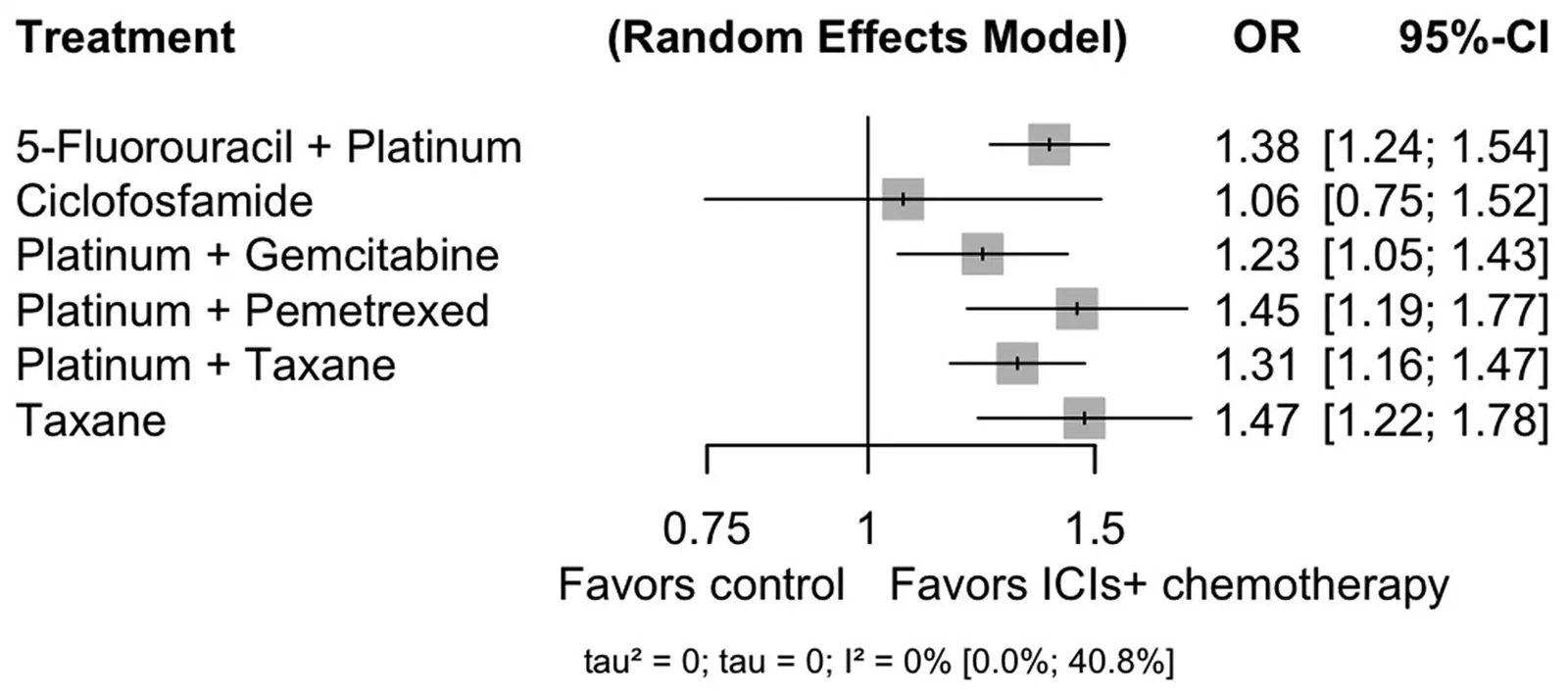
Forest plot of grade ≥3 adverse events stratified by chemotherapy backbone. Forest plot reporting pooled odds ratios for severe toxicities across chemotherapy backbones combined with immune checkpoint inhibitors. Between-study heterogeneity was low (*I*² = 0%). Platinum-pemetrexed (*k* = 4), platinum-taxane (*k* = 10), fluoropyrimidine-platinum (*k* = 11), platinum-gemcitabine (*k* = 5), and taxane-only regimens (*k* = 4) were included in the analysis.

## Discussion

This pan-tumor network meta-analysis identified consistent differences in treatment outcomes across chemotherapy backbones combined with immune checkpoint inhibitors. However, because chemotherapy backbones are closely linked to tumor-specific standards of care, these findings should not be interpreted as evidence that one regimen is intrinsically superior to another across malignancies. Rather, they suggest that chemotherapy backbone may contribute, together with underlying disease biology, to the variability in outcomes observed with chemo-immunotherapy and provide a hypothesis-generating framework for future disease-specific investigations.

The apparent superiority of platinum-pemetrexed and platinum-taxane combinations should be interpreted cautiously because these regimens are predominantly used in tumor types with different intrinsic sensitivity to immune checkpoint inhibition. Nevertheless, although these observations may partly reflect the tumor types in which these regimens are routinely used, preclinical and translational studies offer biological plausibility for a potential contribution of chemotherapy backbone to immunotherapy efficacy. However, biological plausibility should not be interpreted as validation of the associations observed in the present clinical analysis, which cannot disentangle chemotherapy-related effects from the biological characteristics of the tumors in which these regimens are preferentially used. The observed differences may reflect specific immunomodulatory mechanisms, such as immunogenic cell death, enhanced antigen presentation, and facilitation of dendritic-cell maturation and T-cell priming, that appear less pronounced with other cytotoxic agents.^47^^,^^48^ Preclinical evidence supports this interpretation: both anthracyclines and taxanes have been shown to induce robust immunogenic cell stress, ranging from immunogenic modulation to bona fide immunogenic cell death, thereby enhancing tumor antigenicity and sensitivity to immune attack.^49–51^

An important alternative explanation for these findings is that the apparent superiority of certain chemotherapy backbones reflects the intrinsic responsiveness to immune checkpoint inhibitors of the tumor types in which these regimens are predominantly used. This possibility cannot be excluded and represents one of the principal limitations of the present analysis. Accordingly, our findings should be interpreted as suggesting a potential contribution of chemotherapy backbone that cannot be disentangled from underlying disease biology. Although platinum-pemetrexed is predominantly used in non-squamous NSCLC, intra-disease comparisons within lung cancer demonstrated differential gradients between pemetrexed- and taxane-based regimens. However, these observations should not be extrapolated to other malignancies, where tumor biology, patterns of immune responsiveness, and treatment paradigms differ substantially. First, similar directional patterns were observed for taxane-based regimens across several tumor types, including lung, breast, and esophageal cancers. Although this consistency is compatible with a potential contribution of chemotherapy backbone, these indirect observations cannot distinguish chemotherapy-specific effects from disease-specific biological characteristics.^52^^,^^53^ Second, even within the same pathology, such as lung cancer, distinct gradients of efficacy were observed between different backbones. Although these observations are compatible with the hypothesis that chemotherapy backbone may contribute to immune priming, they remain equally compatible with differences in patient populations, disease biology, and other unmeasured sources of confounding. Therefore, they should be regarded as supportive, hypothesis-generating observations rather than evidence of a backbone-specific effect.^54^ Nevertheless, these observations remain hypothesis-generating and do not establish causality.

In contrast, the comparatively modest results observed with platinum–gemcitabine combinations raise important biological considerations. While this may partially reflect the immune-resistant nature of tumors where gemcitabine is commonly used (eg, biliary or urothelial cancers), mechanistic data provide a biologically plausible hypothesis that may partially explain these observations. Gemcitabine is a nucleoside analog with well-documented lymphodepleting properties**:** it inhibits the proliferation of activated T lymphocytes^55^ and depletes dendritic cells and effector subsets.^56^ At the same time, the immunologic effects of gemcitabine appear to be highly dose- and context-dependent. Preclinical studies suggest that low-dose exposure may enhance antitumor immunity through immune priming and depletion of myeloid-derived suppressor cells, whereas conventional cytotoxic dosing may also impair activated T-cell expansion and dendritic-cell function. Therefore, gemcitabine likely exerts both immune-stimulatory and immune-suppressive effects depending on the biological context. Although gemcitabine can reduce myeloid-derived suppressor cells, it may simultaneously impair T-cell expansion and promote M2 macrophage polarization, thereby diminishing the quality of the antitumor immune response.^57^ This contrasts with taxanes, which, despite greater toxicity, consistently induce immunogenic cell stress, increase calreticulin exposure, and enhance NK and CD8+ T-cell cytotoxicity, thereby promoting effective antitumor immunity. Thus, the more favorable safety profile of gemcitabine-based regimens may reflect a lower capacity to generate the antigenic burst and inflammatory milieu required for optimal checkpoint inhibitor activity. Importantly, gemcitabine-based regimens still demonstrated clinically meaningful benefit compared with chemotherapy alone, although the magnitude of benefit appeared lower relative to other backbones.

Anti–PD-1 agents showed numerically greater pooled effect sizes compared with anti–PD-L1 therapies; however, this difference was not statistically confirmed in meta-regression analyses and may partly reflect differences in tumor-type representation across trials. While direct head-to-head comparisons remain scarce, the cross-tumor consistency observed here strengthens the biological plausibility of this difference. Tumor-intrinsic interferon signaling, antigen presentation machinery, and T-cell reinvigoration thresholds, processes influenced by chemotherapy-induced immunogenic cell stress, could potentially differentially interact with the pharmacology of PD-1 vs PD-L1 inhibitors.^58^ Although exploratory, this observation deserves further investigation because it remained directionally consistent across several subgroup analyses despite the absence of a statistically significant interaction in meta-regression. Whether this reflects biological differences between PD-1 and PD-L1 blockade, differences in trial populations, or residual confounding cannot be determined from the present analysis.

Perhaps the most clinically relevant finding is that chemotherapy backbone selection may represent a potentially modifiable component of chemo-immunotherapy optimization within established disease-specific standards of care. While tumor biology remains central to immunotherapy responsiveness, this analysis suggests that the therapeutic context in which ICIs are delivered, including chemotherapy backbone, may contribute to differences in clinical outcomes. As such, future clinical trials evaluating new immunotherapy agents or combination regimens may benefit from considering chemotherapy partners supported by preclinical evidence of immunologic synergy, ideally those capable of inducing sustained immunogenic cell stress, enhancing antigenicity, and modulating the tumor microenvironment toward effective T-cell engagement.

A central challenge in interpreting the present findings lies in the close relationship between chemotherapy backbone and tumor type. In current clinical practice, chemotherapy regimens are not randomly assigned across malignancies but represent disease-specific standards of care developed over decades of clinical investigation. Consequently, differences observed between chemotherapy backbones inevitably reflect not only the characteristics of the cytotoxic agents themselves but also the biological context in which those regimens are used, including intrinsic tumor immunogenicity, sensitivity to immune checkpoint inhibition, and disease-specific treatment paradigms. Although disease-specific analyses and meta-regression were performed to explore the robustness of these observations, these approaches cannot fully overcome this structural limitation of aggregate trial-level comparisons. Moreover, because chemotherapy backbones are inherently embedded within disease-specific treatment paradigms, it is not possible within the present study design to disentangle the relative contribution of chemotherapy-related immunomodulatory effects from the intrinsic biological characteristics of the tumors in which these regimens are preferentially used. Consequently, the observed differences should be interpreted as associations rather than evidence of causality. Therefore, the present analysis should not be interpreted as demonstrating the superiority of one chemotherapy backbone over another across tumor types. Rather, it provides a framework for identifying recurring patterns that may justify prospective evaluation within individual malignancies, where the independent contribution of chemotherapy backbone can be assessed under substantially more homogeneous biological conditions.

This study has several limitations. First, this network meta-analysis relies on published aggregate data rather than individual patient data, which may introduce ecological bias and preclude adjustment for important patient-level characteristics such as PD-L1 expression, tumor mutational burden, and other potential confounders. Second, although cross-tumor comparisons provide valuable exploratory insights, heterogeneity in trial design, patient selection, chemotherapy dosing schedules, and supportive treatment strategies may have influenced the observed treatment effects. Furthermore, because chemotherapy backbone and tumor type were intrinsically linked across the included trials, the assumption of transitivity underlying network meta-analysis may have been only partially satisfied despite the overall clinical similarity of the included populations. Third, toxicity analyses relied on reported grade ≥3 adverse events, which were not uniformly defined across studies. Finally, several backbone categories included relatively few studies, limiting precision, while clinically relevant differences within backbone groups—including drug dose, scheduling, maintenance strategies, and partner agents—could not be explored using aggregate published data. In addition, potentially relevant factors such as maintenance strategies, treatment duration, PD-L1 enrichment, and regional differences in trial populations may also have contributed to residual heterogeneity that could not be adequately accounted for in the present analysis. Taken together, these limitations emphasize that the present findings should primarily be viewed as hypothesis-generating rather than practice-changing. Accordingly, our findings are intended to inform future hypothesis-driven research rather than to support changes in current clinical practice. Nevertheless, the consistency of the observed patterns across multiple randomized trials supports further prospective and translational investigations aimed at determining whether chemotherapy backbone exerts an independent contribution to immunotherapy efficacy within individual tumor types.

## Conclusions

This comprehensive pan-tumor analysis identified consistent differences in outcomes across chemotherapy backbones used in combination with immune checkpoint inhibitors within tumor-specific standards of care. Associations between chemotherapy backbone and the relative efficacy of chemo-immunotherapy were observed across the included randomized trials. Platinum–pemetrexed and platinum–taxane regimens were associated with numerically greater relative benefit, whereas platinum–gemcitabine combinations showed comparatively lower benefit. However, these associations cannot be disentangled from tumor-specific biology or treatment paradigms and therefore should not be interpreted as evidence of intrinsic superiority of any chemotherapy backbone across tumor types.

In contrast, platinum–gemcitabine-based regimens showed less robust benefit. The apparent superiority of anti–PD-1 over anti–PD-L1 agents suggests biological relevance, although this observation is influenced by differential tumor representation across trials.

These results should not be interpreted as evidence of full cross-tumor interchangeability of chemotherapy backbones. Chemotherapy selection is closely linked to tumor biology and established treatment paradigms. The observed associations likely may reflect both backbone-specific effects and underlying differences in tumor immunogenicity. Any apparent consistency across tumor types should therefore be viewed as directional rather than quantitatively uniform.

Taken together, this analysis indicates that chemotherapy backbone selection may represent one of several factors contributing to the therapeutic context in which immunotherapy is delivered. Optimizing backbone choice within tumor-specific standards of care, rather than assuming equivalence across cytotoxic regimens, may represent a pragmatic strategy to maximize the therapeutic potential of ICIs.

Future prospective and translational studies performed within individual tumor types will be essential to determine whether chemotherapy backbone exerts an independent immunologic contribution beyond the underlying biology of the diseases in which these regimens are used.

## Supplementary Material

oyag365_Supplementary_Data

## Data Availability

All data analyzed in this study were derived from previously published studies and are included in the article and its Supplementary Material. Additional analytical details are available from the corresponding author upon reasonable request.
