# Supplementary material for: Chemotherapy backbone and outcomes of immune checkpoint inhibitor combinations across solid tumors: a pan-tumor network meta-analysis
Source: Oncologist. 2026 Sep 17;31(11):oyag365. doi: 10.1093/oncolo/oyag365 (PMC13652018; doi:10.1093/oncolo/oyag365)
Supplement: Supplementary file 1 [file oyag365_supplementary_data.zip › Supplementary_Data/Supplementary Figures.docx]

# Supplementary Figure S1.

**Supplementary Figure S1. Risk of bias (RoB 2)** Risk-of-bias assessment of included randomized clinical trials using the Cochrane Risk of Bias 2 (RoB 2) tool. Most studies were judged to have a low risk of bias across all assessed domains, with a limited number showing some concerns, mainly related to missing outcome data or outcome measurement. D1, bias arising from the randomization process; D2, bias due to deviations from intended interventions; D3, bias due to missing outcome data; D4, bias in measurement of the outcome; D5, bias in selection of the reported result. Judgment: green = low risk of bias; yellow = some concerns.

# Supplementary Figure S2.

**Supplementary Figure S2. Forest plot of progression-free survival (PFS) with first-line chemo-immunotherapy across all included trials.** The plot displays individual and pooled hazard ratios (HRs) comparing chemo-immunotherapy versus chemotherapy alone. Overall, chemo-immunotherapy was associated with a significant improvement in PFS compared with chemotherapy alone (pooled HR, 0.69; 95% CI, 0.66–0.72; p < 0.0001). Heterogeneity across studies was moderate (I² = 54.0%). Abbreviations: PFS, progression-free survival; HR, hazard ratio; CI, confidence interval; ICI, immune checkpoint inhibitor.

# Supplementary Figure S3.

**Supplementary Figure S3. Forest plot of progression-free survival stratified by chemotherapy backbone.** The plot compares pooled hazard ratios (HRs) for PFS across predefined chemotherapy backbones combined with immune checkpoint inhibitors (ICIs). Platinum-pemetrexed–based regimens were associated with the greatest improvement in PFS, followed by taxane-containing combinations. Overall heterogeneity across subgroups was moderate (I² = 58.5%). Abbreviations: PFS, progression-free survival; HR, hazard ratio; CI, confidence interval; ICI, immune checkpoint inhibitor

# Supplementary Figure S4.

**Supplementary Figure S4. Forest plot of the different chemo-ICI combinations**: The plot displays pooled hazard ratios (HRs) for PFS across different chemotherapy backbones combined with immune checkpoint inhibitors (ICIs), consistently favoring chemo-immunotherapy over chemotherapy alone. Platinum-pemetrexed– and taxane-based regimens showed the greatest magnitude of benefit. Overall heterogeneity across subgroups was moderate (I² = 59.7%). Abbreviations: PFS, progression-free survival; HR, hazard ratio; CI, confidence interval; ICI, immune checkpoint inhibitor.

# Supplementary Figure S5.

**Supplementary Figure S5. Forest plot of overall survival (OS) according to different chemo-immunotherapy combinations stratified by anti–PD-1 and anti–PD-L1 regimens**. The plot displays pooled hazard ratios (HRs) for OS across different chemotherapy backbones combined with immune checkpoint inhibitors (ICIs). Most combinations favored chemo-immunotherapy over chemotherapy alone, with the greatest magnitude of benefit observed for platinum-pemetrexed–based regimens. Overall heterogeneity across subgroups was low to moderate (I² = 35.0%). Abbreviations: OS, overall survival; HR, hazard ratio; CI, confidence interval; PD-1, programmed cell death protein 1; PD-L1, programmed death-ligand 1; ICI, immune checkpoint inhibitor.

# Supplementary Figure S6.

**Supplementary Figure S6. Forest plot of progression-free survival stratified by tumor type and chemotherapy backbone.** The plot displays pooled hazard ratios (HRs) for PFS across individual tumor types according to the associated chemotherapy backbone combined with immune checkpoint inhibitors (ICIs). Platinum-pemetrexed–based regimens in lung cancer and taxane-containing regimens across multiple tumor types were associated with the greatest magnitude of benefit. Overall heterogeneity across subgroups was moderate (I² = 61.1%). Abbreviations: PFS, progression-free survival; HR, hazard ratio; CI, confidence interval; ICI, immune checkpoint inhibitor.

# Supplementary Figure S7.

**Supplementary Figure S7. Forest plot of Overall survival stratified by tumor type and chemotherapy backbone.** The plot displays pooled hazard ratios (HRs) for OS across individual tumor types according to the associated chemotherapy backbone combined with immune checkpoint inhibitors (ICIs). Platinum-pemetrexed–based regimens in lung cancer and fluorouracil-platinum combinations in gastrointestinal tumors were associated with the greatest magnitude of survival benefit. Overall heterogeneity across subgroups was low to moderate (I² = 33.7%). Abbreviations: OS, overall survival; HR, hazard ratio; CI, confidence interval; ICI, immune checkpoint inhibitor.

# Supplementary Figure S8.

**Supplementary Figure S8. Forest plot of Progression-free survival comparing anti–PD1 versus anti–PDL1 regimens.** The plot displays pooled hazard ratios (HRs) for PFS associated with anti–PD-1 and anti–PD-L1 agents combined with chemotherapy. Both treatment classes were associated with improved PFS compared with chemotherapy alone, with numerically greater benefit observed for anti–PD-1–based regimens. Overall heterogeneity across subgroups was moderate (I² = 61.6%). Abbreviations: PFS, progression-free survival; HR, hazard ratio; CI, confidence interval; PD-1, programmed cell death protein 1; PD-L1, programmed death-ligand 1; ICI, immune checkpoint inhibitor.

# Supplementary Figure S9.

# Supplementary Figure S9. Forest plot of Overall survival comparing anti–PD1 versus anti–PDL1 regimens. The plot displays pooled hazard ratios (HRs) for OS associated with anti–PD-1 and anti–PD-L1 agents combined with chemotherapy. Both treatment classes were associated with improved OS compared with chemotherapy alone, with similar magnitude of benefit across subgroups. Overall heterogeneity across subgroups was moderate (I² = 41.2%). Abbreviations: OS, overall survival; HR, hazard ratio; CI, confidence interval; PD-1, programmed cell death protein 1; PD-L1, programmed death-ligand 1; ICI, immune checkpoint inhibitor.

# Supplementary Figure S10.

**Supplementary Figure S10. Forest plot of OR of Grade ≥3 adverse events stratified by tumor type.** The plot displays pooled ORs for grade ≥3 treatment-related toxicities across individual tumor types treated with chemo-immunotherapy versus chemotherapy alone. Most tumor types showed a higher incidence of severe adverse events with chemo-immunotherapy combinations compared with chemotherapy alone. No substantial heterogeneity across subgroups was observed (I² = 0%). Abbreviations: OR, odds ratio; CI, confidence interval; ICI, immune checkpoint inhibitor.

# Supplementary Figure S11.

**Supplementary Figure S11.** The plot displays pooled ORs comparing the toxicity profiles of anti–PD-1 and anti–PD-L1 agents combined with chemotherapy. Both treatment classes were associated with an increased incidence of grade ≥3 adverse events compared with chemotherapy alone, with similar toxicity profiles across subgroups. No substantial heterogeneity across subgroups was observed (I² = 0%). Abbreviations: OR, odds ratio; CI, confidence interval; PD-1, programmed cell death protein 1; PD-L1, programmed death-ligand 1; ICI, immune checkpoint inhibitor.

# Supplementary Figure S12.

**Supplementary Figure S12. Meta-regression assessing tumor-type effects**. No tumor type was identified as a significant moderator of treatment effect, and confidence intervals were generally wide, indicating substantial uncertainty in subgroup estimates. Abbreviations: SE, standard error; CI, confidence interval.

# Supplementary Figure S13.

**Supplementary Figure S13. Meta-regression including ICI class (anti–PD-L1) as covariate.** The analysis did not identify ICI class as a significant moderator of treatment effect size. Abbreviations: ICI, immune checkpoint inhibitor; PD-L1, programmed death-ligand 1; SE, standard error; CI, confidence interval.
