# Supplementary material for: Chemotherapy backbone and outcomes of immune checkpoint inhibitor combinations across solid tumors: a pan-tumor network meta-analysis
Source: Oncologist. 2026 Sep 17;31(11):oyag365. doi: 10.1093/oncolo/oyag365 (PMC13652018; doi:10.1093/oncolo/oyag365)
Supplement: Supplementary file 1 [file oyag365_supplementary_data.zip › Supplementary_Data/Supplementary Tables[AU].docx]

**Supplementary Table S1. Lung Cancer Trials**

**Characteristics and outcomes of lung cancer randomized trials included in the meta-analysis**

| **Study** | **Setting** | **Immunotherapy** | **Chemotherapy backbone** | **N Exp** | **N Ctrl** | **HR PFS** | **95% CI PFS** | **HR OS** | **95% CI OS** | **G3 AEs Exp** | **G3 AEs Ctrl** |
| --- | --- | --- | --- | --- | --- | --- | --- | --- | --- | --- | --- |
| Melissa L. Johnson, 2022a | 1st line | Durvalumab | Platinum + Pemetrexed (with maintenance) | 214 | 214 | 0.77 | 0.61–0.96 | 0.86 | 0.72–1.02 | 0.450 | 0.440 |
| Melissa L. Johnson, 2022b | 1st line | Durvalumab | Platinum + Gemcitabine / Platinum + Paclitaxel | 128 | 122 | 0.68 | 0.52–0.90 | 0.84 | 0.64–1.10 | 0.450 | 0.440 |
| Silvia Novello, 2023 | 1st line | Pembrolizumab | Carboplatin + Paclitaxel / nab-Paclitaxel | 278 | 281 | 0.62 | 0.52–0.74 | 0.71 | 0.59–0.85 | 0.748 | 0.700 |
| David P. Carbone, 2024 (entry 1) | 1st line | Nivolumab | (not specified in file) | 555 | 557 | 0.75 | 0.65–0.87 | 0.73 | 0.62–0.86 | 0.507 | 0.559 |
| Makoto Nishio, 2021 (IMpower132) | 1st line | Atezolizumab | Carboplatin + Pemetrexed | 292 | 286 | 0.60 | 0.49–0.72 | 0.76 | 0.63–0.94 | 0.663 | 0.671 |
| Delvys Rodríguez-Abreu, 2021 (KEYNOTE-024/042 subset) | 1st line | Pembrolizumab | Platinum-based | 637 | 637 | 0.68 | 0.57–0.80 | 0.73 | 0.61–0.88 | 0.630 | 0.600 |
| Howard West, 2019 (IMpower130) | 1st line | Atezolizumab | Carboplatin + nab-Paclitaxel | 357 | 359 | 0.64 | 0.54–0.76 | 0.78 | 0.64–0.96 | 0.692 | 0.701 |
| Robert Jotte, 2020 (IMpower131) | 1st line | Atezolizumab | Carboplatin + Paclitaxel/nab-Paclitaxel | 343 | 340 | 0.72 | 0.60–0.86 | 0.88 | 0.73–1.07 | 0.726 | 0.710 |
| Mark Awad, 2021 (CheckMate 9LA) | 1st line | Nivolumab + Ipilimumab | Platinum doublet | 361 | 358 | 0.68 | 0.58–0.81 | 0.74 | 0.62–0.89 | 0.490 | 0.450 |

**Supplementary Table S2. Breast Cancer Trials**

**Characteristics and outcomes of breast cancer randomized trials included in the meta-analysis**

| **Study** | **Setting** | **Immunotherapy** | **Chemotherapy backbone** | **N Exp** | **N Ctrl** | **HR PFS** | **95% CI PFS** | **HR OS** | **95% CI OS** | **G3 AEs Exp** | **G3 AEs Ctrl** |
| --- | --- | --- | --- | --- | --- | --- | --- | --- | --- | --- | --- |
| Andresen 2024 | 1st/2nd line | Ipilimumab + Nivolumab | PLD + Cyclophosphamide | 49 | 33 | 0.94 | 0.59–1.51 | NA | NA | 24% | 48% |
| Cortes 2022 | 1st line | Pembrolizumab | Nab-paclitaxel / Paclitaxel / Gemcitabine + Carboplatin | 566 | 281 | 0.66 | 0.50–0.88 | 0.73 | 0.55–0.95 | 68.1% | 66.9% |
| Miles 2021 | 1st line | Atezolizumab | Paclitaxel | 431 | 220 | 0.82 | 0.60–1.12 | 1.11 | 0.76–1.64 | 53% | 46% |
| Brufsky 2021 | 1st line | Atezolizumab | Cobimetinib + Paclitaxel | 47 | 32 | 0.73 | 0.43–1.24 | 1.05 | 0.55–2.01 | 36% | 47% |
| Dent 2024 | 1st line | Atezolizumab | Carboplatin + Gemcitabine / Capecitabine | 177 | 177 | 0.84 | 0.67–1.06 | 0.93 | 0.73–1.20 | 67% | 71% |
| Schmid 2020 | 1st line | Atezolizumab | Nab-paclitaxel | 451 | 451 | 0.63 | 0.50–0.80 | 0.71 | 0.54–0.94 | 49% | 43% |
| Buisseret 2023 | 1st line | Durvalumab ± Oleclumab | Paclitaxel + Carboplatin | 63 | 64 | 0.87 | 0.55–1.37 | 1.11 | 0.66–1.87 | 15.9% | 12.5% |
| Jiang 2024 | 1st/2nd line | Toripalimab | Nab-paclitaxel | 353 | 178 | 0.65 | 0.47–0.91 | 0.62 | 0.41–0.91 | 56.1% | 54.3% |

**Supplementary Table S3. Gynecologic Cancer Trials**

**Characteristics and outcomes of gynecologic cancer randomized trials included in the meta-analysis**

| **Study** | **Setting** | **Immunotherapy** | **Chemotherapy backbone** | **N Exp** | **N Ctrl** | **HR PFS** | **95% CI PFS** | **HR OS** | **95% CI OS** | **G3 AEs Exp** | **G3 AEs Ctrl** |
| --- | --- | --- | --- | --- | --- | --- | --- | --- | --- | --- | --- |
| Pignata et al., 2023 | 1st line Endometrial | Avelumab | Carboplatin + Paclitaxel | 63 | 62 | 0.64 | 0.44–0.93 | 0.83 | 0.56–1.24 | — | — |
| Colombo et al., 2024 | 1st line Endometrial | Atezolizumab | Carboplatin AUC 5/6 + Paclitaxel 175 mg/m² | 360 | 189 | 0.78 | 0.65–0.94 | 0.90 | 0.73–1.12 | — | — |
| Mansoor R. Mirza, 2023 | 1st line Endometrial | Dostarlimab | Carboplatin + Paclitaxel | 245 | 249 | 0.64 | 0.51–0.80 | 0.79 | 0.62–1.01 | — | — |
| Eskander et al., 2023 | 1st line Endometrial | Pembrolizumab | Carboplatin + Paclitaxel | 405 | 399 | 0.54 | 0.46–0.64 | 0.64 | 0.52–0.79 | — | — |

**Supplementary Table S4. Gastrointestinal Cancer Trials**

**Characteristics and outcomes of gastrointestinal cancer randomized trials included in the meta-analysis**

| **Study** | **Setting** | **Immunotherapy** | **Chemotherapy backbone** | **N Exp** | **N Ctrl** | **HR PFS** | **95% CI PFS** | **HR OS** | **95% CI OS** | **G3 AEs Exp** | **G3 AEs Ctrl** |
| --- | --- | --- | --- | --- | --- | --- | --- | --- | --- | --- | --- |
| Boku 2024 | 1st line | Nivolumab | SOX / CAPOX | 362 | 362 | 0.68 | 0.53–0.87 | 0.80 | 0.62–1.04 | — | — |
| Miao-Zhen Qiu | 1st line | Tislelizumab | CAPOX / Cis + 5-FU | 501 | 496 | 0.73 | 0.60–0.89 | 0.89 | 0.73–1.10 | — | — |
| Xu et al., 2023 | 1st line | Sintilimab | XELOX | 327 | 323 | 0.57 | 0.45–0.72 | 0.70 | 0.56–0.88 | — | — |
| Zhang et al., 2025 | 1st line | Sugemalimab | CAPOX | 241 | 238 | 0.69 | 0.54–0.89 | 0.84 | 0.64–1.12 | — | — |
| Shitara et al., 2020 | 1st line | Pembrolizumab | Cisplatin + 5-FU / Capecitabine | 257 | 250 | 0.73 | 0.63–0.86 | 0.91 | 0.76–1.09 | — | — |
| Sun et al., 2021 | 1st line | Pembrolizumab | Cisplatin + 5-FU | 373 | 376 | 0.65 | 0.55–0.76 | 0.73 | 0.62–0.86 | — | — |
| Rha et al., 2023 | 1st line | Pembrolizumab | Cisplatin + 5-FU / CAPOX | 790 | 789 | 0.76 | 0.67–0.85 | 0.78 | 0.70–0.87 | — | — |
| Lin Shen et al., 2025 | 1st line | Cadonilimab | XELOX | 305 | 305 | 0.53 | 0.44–0.65 | 0.66 | 0.54–0.81 | — | — |

**Supplementary Table S5. Urogenital Cancer Trials**

**Characteristics and outcomes of urothelial cancer randomized trials included in the meta-analysis**

| **Study** | **Setting** | **Immunotherapy** | **Chemotherapy backbone** | **N Exp** | **N Ctrl** | **HR PFS** | **95% CI PFS** | **HR OS** | **95% CI OS** | **G3 AEs Exp** | **G3 AEs Ctrl** |
| --- | --- | --- | --- | --- | --- | --- | --- | --- | --- | --- | --- |
| Powles et al., 2021 | 1st line | Pembrolizumab | Cisplatin/Carboplatin + Gemcitabine | 351 | 352 | 0.65 | 0.55–0.76 | 0.79 | 0.66–0.94 | — | — |
| van der Heijden et al., 2023 | 1st line | Nivolumab | Cisplatin + Gemcitabine | 304 | 304 | 0.70 | 0.58–0.85 | 0.84 | 0.69–1.01 | — | — |
| Grande et al., 2024 | 1st line | Atezolizumab | Cisplatin/Carboplatin + Gemcitabine | 451 | 400 | 0.73 | 0.60–0.90 | 0.85 | 0.73–1.00 | — | — |

**Supplementary Table S6. Esophageal Squamous Cell Carcinoma Trials**

**Characteristics and outcomes of esophageal squamous cell carcinoma randomized trials included in the meta-analysis**

| **Study** | **Setting** | **Immunotherapy** | **Chemotherapy backbone** | **N Exp** | **N Ctrl** | **HR PFS** | **95% CI PFS** | **HR OS** | **95% CI OS** | **G3 AEs Exp** | **G3 AEs Ctrl** |
| --- | --- | --- | --- | --- | --- | --- | --- | --- | --- | --- | --- |
| Zi-Xian Wang, 2024 | 1st line | Toripalimab | Paclitaxel + Cisplatin | 257 | 257 | 0.58 | 0.47–0.72 | 0.70 | 0.55–0.88 | 0.988 | 0.992 |
| Kato et al., 2024 | 1st line | Nivolumab | Cisplatin + 5-FU | 321 | 324 | 0.76 | 0.64–0.90 | 0.81 | 0.68–0.96 | 0.990 | 0.990 |
| Luo et al., 2021 | 1st line | Camrelizumab | Cisplatin + Paclitaxel | 298 | 298 | 0.69 | 0.58–0.83 | 0.76 | 0.63–0.92 | 0.993 | 0.987 |
| Yan Song, 2023 | 1st line | Serplulimab | Cisplatin + 5-FU | 368 | 368 | 0.66 | 0.56–0.77 | 0.74 | 0.61–0.89 | 0.993 | 0.981 |
